# Preoperative prealbumin levels on admission as an independent predictive factor in patients with gastric cancer

**DOI:** 10.1097/MD.0000000000019196

**Published:** 2020-03-13

**Authors:** Hongliang Zu, Huiling Wang, Chunfeng Li, Yingwei Xue

**Affiliations:** a Department of Gastroenterologic Surgery; b Department of ICU, The First People's Hospital of Zhaoqing, Zhaoqing City, Guangdong Province; c Department of Gastroenterologic Surgery, Affiliated Tumor Hospital of Harbin Medical University, Harbin, Heilongjiang Province, China.

**Keywords:** gastric cancer, prealbumin, prognosis

## Abstract

**Background:**

To explore the role of preoperative prealbumin levels in predicting the prognosis of patients with gastric cancer.

**Methods:**

A total of 989 gastric cancer patients in the Affiliated Tumour Hospital of Harbin Medical University who underwent gastrectomy were included in this retrospective study. The preoperative prealbumin level, clinicopathological data, and follow-up data were recorded. According to the maximum chi-square survival correlation value, the survival of patients with low preoperative prealbumin (<140 mg/L) and high preoperative prealbumin (≥140 mg/L) were compared using the log-rank test and the Cox proportional hazard regression model.

**Results:**

Based on the best cut-off value of 140 mg/L, we divided the patients into the lower prealbumin group (<140 mg/L) and the higher prealbumin group (≥140 mg/L). Compared with the higher prealbumin group, the lower prealbumin group were older and had larger tumor volumes, lower hemoglobin (Hb) levels, and more upper gastric cancer tumors. The univariate analysis showed that prealbumin and other clinicopathological factors, including age, hemoglobin, tumor size, macroscopic type, cell differentiation, liver metastasis, operation type, N stage, and T stage, were significant prognostic factors. The multivariable analysis showed that age, prealbumin, macroscopic type, location, T stage, and N stage were independent prognostic factors.

**Conclusions:**

The preoperative prealbumin level was an independent prognostic factor for patients with gastric cancer. The preoperative prealbumin level can be used to predict the prognosis of patients with gastric cancer and guide clinical practice.

## 1 Introduction

Gastric cancer (GC) is one of the most common cancers and is the third most frequent cause of mortality worldwide.^[1]^ Notably, half of all GCs occur in Eastern Asia (mainly in China); China alone represents almost 42% of the world's GC burden and 45.0% of the worldwide deaths.^[2]^ GC patients are often diagnosed in advanced stages in China, and the 5-year survival rate of patients with advanced disease remains poor, at approximately ≤20%.^[2]^ Therefore, it is necessary to improve the prognosis of this disease. At present, the TNM staging system is the most common method to judge the prognosis of GC patients.^[3]^ This system relies on surgical specimens, which cannot be acquired before surgery. Prealbumin has a half-life of approximately 2 days and is readily available clinical material. It is highly sensitive to many diseases and has been paid an increasing amount of attention in recent years. Preoperative prealbumin is an independent prognostic factor for many malignant diseases^[4–7]^ and was found to be an independent prognostic factor in patients undergoing gastrectomy for stage II/III GC,^[8]^ but there are few reports of patients at all stages. In this study, the preoperative prealbumin levels and clinicopathological data of 989 patients with GC were analyzed retrospectively to explore the prognostic value of preoperative prealbumin for GC.

## 2 Patients and methods

### 2.1 Patients

A total of 989 GC patients from the Department of Surgical Gastroenterology, Affiliated Tumour Hospital of Harbin Medical University between 2008 and 2010 were reviewed retrospectively. This study was approved by the Ethics Committee of the Harbin Medical University, and all patients provided informed consent before enrolment in the study. These patients had primary gastric adenocarcinoma that was histologically proven by endoscopic biopsy before surgery. GC was further confirmed by histopathology after surgery. None of these patients received preoperative chemoradiotherapy. Patients with multiple primary cancers accompanied by hepatic disease, chronic renal disease, thyroid disorders, or other diseases that could induce variations in their prealbumin levels were excluded from this study. To determine an optimal prealbumin threshold, the survival rates were evaluated in 10 mg/L intervals. The survival rates were compared with the established threshold by the log-rank test and Cox proportional hazard model. The threshold prealbumin value was identified as the test size with the maximum chi-square (*χ*^2^) value (as shown in Table 1). The largest chi-square value was associated with a disease-specific survival of 49.966 (*P* < .001, 95% CI = [39.274–58.726]) in the log-rank test and 18.349 (*P* = .000, 95% CI = [0.388–0.703]) in the Cox proportional hazard model. Then, we determined 140 mg/L as the optimal prealbumin cut-off value. A total of 989 consecutive GC patients were divided into 2 groups: <140 mg/L (n = 62) and ≥140 mg/L (n = 927). The following clinicopathological data were collected: sex (male or female), age (years, mean, SD), Hb level (g/L, mean, SD), prealbumin level (mg/L, mean, SD), tumor size (cm, mean, SD), macroscopic type (Borrmann I, II, III, IV, or X), cell differentiation (well-differentiated, moderately differentiated, poorly differentiated, mucinous carcinoma, or signet ring cell carcinoma; if there were ≥2 histological types, the histological type was defined as the predominant type in the tumor), tumor location (upper, middle, lower, or whole), and surgical extent (radical or non-radical). Regarding the surgical extent (radical, non-radical), surgery was deemed radical when there was no residual tumor (R0 resection); otherwise, the surgery was considered non-radical (R1 or R2).^[9]^ Our study recorded laparotomy and bypass (gastrojejunostomy) procedures and collected liver and peritoneal metastases. The 7th American Joint Committee on Cancer (AJCC) lymph node status (N0, N1, N2, N3a, N3b) and depth of tumor invasion (T1: tumor has invaded the mucosa or submucosa layer; T2: tumor has invaded the muscular layer or the subserosa; T3: tumor has invaded the subserosa; T4a: tumor has invaded the serosa or has penetrated the serosa; and T4b: tumor has the invaded adjacent organs) classifications were used.^[10]^

**Table 1:**
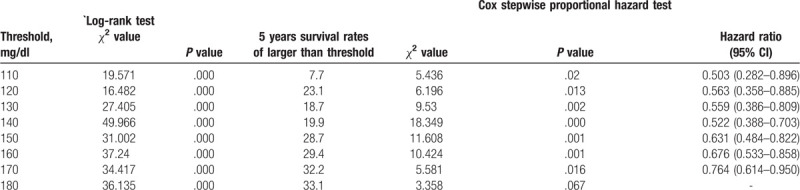
Chi-square value and 5-year survival by prealbumin calculated by log-rank test and Cox stepwise proportional hazard test.

### 2.2 Follow-up and statistics

The patients were followed up until death or the cut-off date of June 31, 2017; the longest period of follow-up was 104 months (median: 44.3 months, range: 0.3–104 months). For patients who survived, the data were censored at the last date of contact. Tumor-related deaths were only classified as patients who died of GC. Continuous variables were expressed as the means ± standard deviations and were compared by the *t* test. Chi-square and Fisher exact tests were used to analyze the associations between categorical variables. The Kaplan–Meier method was used to calculate the overall survival rate, and the log-rank test was employed to compare the survival rates between the lower and higher prealbumin groups. The factors found to be significant in the univariate analysis were entered into the multivariate analysis with a Cox proportional hazard model to identify the independent predictors of prognosis. The criterion for statistical significance was *P* < .05. All data analyses were performed using SPSS for Windows, version 22.0 software (SPSS Inc., Chicago, IL).

## 3 Results

### 3.1 Cut-off value of prealbumin

The largest chi-square value was associated with a disease-specific survival of 49.966 (*P* < .001, 95% CI = [39.274–58.726]) in the log-rank test and 18.349 (*P* = .000, 95% CI = [0.388–0.703]) in the Cox stepwise proportional hazard analysis (as shown in Table 1). A preoperative prealbumin level of 140 mg/L was determined as the optimal cut-off value. A total of 989 consecutive GC patients were divided into the lower prealbumin group (<140 mg/L) and the higher prealbumin group (≥140 mg/L).

### 3.2 Clinicopathologic features of GC according to the preoperative prealbumin levels

Table 2 shows the division of the 989 patients diagnosed with GC into the lower prealbumin group (n = 62) and the higher prealbumin group (n = 927). The mean age of the lower prealbumin group was 61.50 ± 13.95 years old, which was older than the 57.64 ± 11.19 years of the higher prealbumin group (*P* = .01). Moreover, the lower prealbumin group had a lower Hb level (102.45 ± 28.06 g/L vs 127.79 ± 26.09 g/L, *P* = .000), larger tumor size (7.24 ± 4.40 cm vs 5.53 ± 3.63 cm, *P* = .004), and more upper tumors (22.6% vs 10.7%, *P* = .032) than the higher prealbumin group. With regard to the depth of tumor invasion, the lower prealbumin group had more T4a (53.2% vs 40.9%) and T4b (24.2% vs 14.1%) lesions, fewer T1 (0% vs 12.58%) and T2 (4.8% vs 14.8%) lesions and more N3a (24.2% vs 18.1%) and N3b (12.9% vs 7.8%) lesions than the higher prealbumin group. The patients in the lower prealbumin group underwent fewer radical gastrectomy procedures than those in the higher prealbumin group. There were no differences in sex, macroscopic type, or peritoneal metastasis between the 2 groups.

**Table 2:**
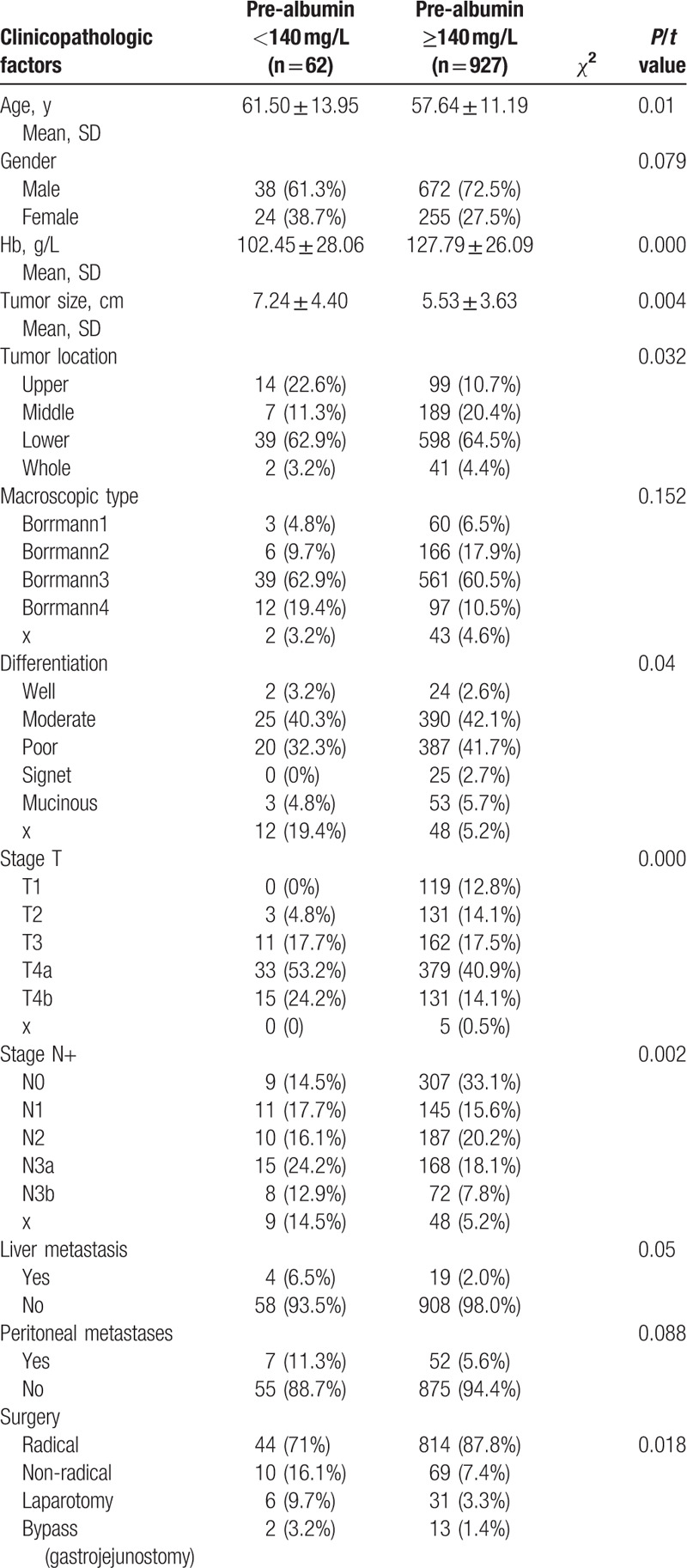
Clinicopathologic features of gastric patients according to preoperative prealbumin levels.

### 3.3 Univariate and multivariable survival analyses

**Figure 1:**
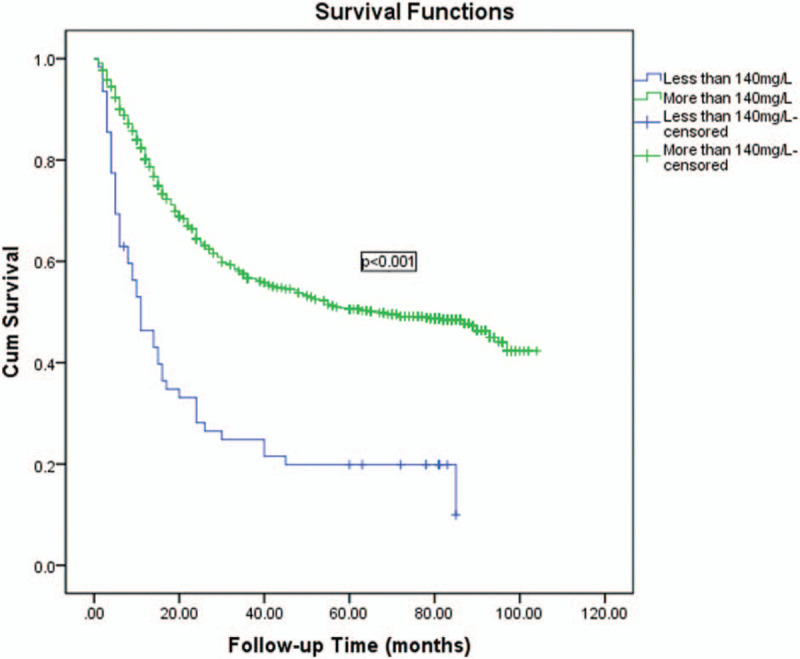
Kaplan–Meier curves of survival indicate a significantly lower 5-year survival rate in the lower prealbumin group than in the higher prealbumin group (the figure includes non-radical resection, n = 989).

**Figure 2:**
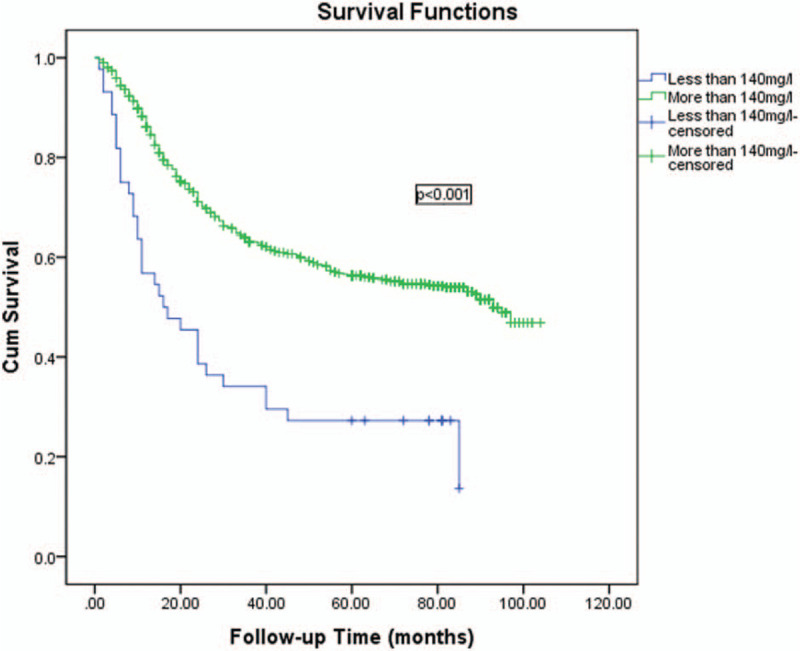
Kaplan–Meier curves of survival indicate a significantly lower 5-year survival rate in the lower prealbumin group than in the higher prealbumin group (the figure includes only radical resection, n = 857).

Univariate and multivariate analyses were used to assess the related clinicopathological variables (Tables 3 and 4). The univariate analysis showed that the prealbumin and other clinicopathological parameters, including age, Hb, tumor size, macroscopic type, cell differentiation, liver metastasis, operation type, N stage, and T stage were significant prognostic factors. Figures 1 and 2 show the Kaplan–Meier curves of survival, which indicate a significantly lower 5-year survival rate in the lower prealbumin group than in the higher prealbumin group (Fig. 1 includes non-radical resection and Fig. 2 includes only patients who underwent radical resection). After adjusting for the potential confounders (factors with *P*-values ≤.05 in the univariate analysis), the multivariable analysis showed that age, prealbumin, macroscopic type, tumor location, T stage, and N stage were independent prognostic factors in all 989 patients (n = 989, as shown in Table 4). The prealbumin level was also an independent prognostic factor for patients who underwent radical resection (n = 857, as shown in Table 5).

**Table 3:**
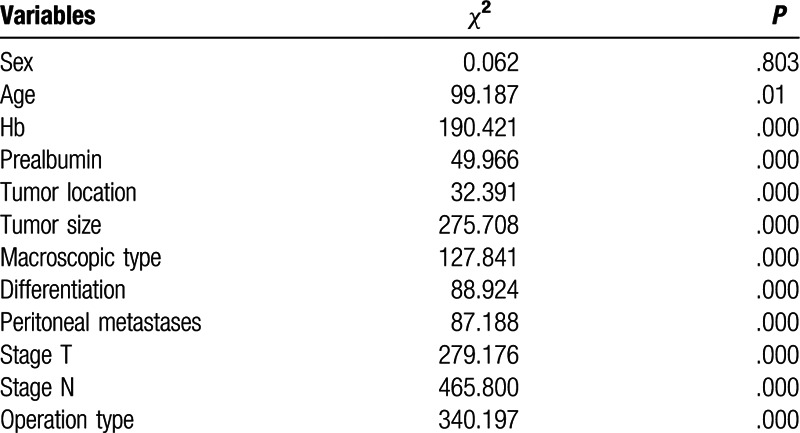
Univariate analysis showed that prealbumin and other clinicopathological parameters, were significant prognostic factors.

**Table 4:**
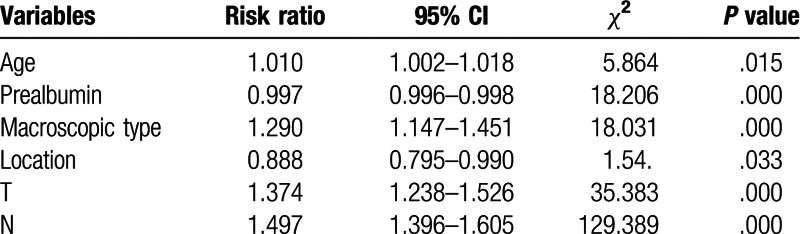
Multivariate analysis of prognostic factors in 989 patients with gastric cancer (n = 989).

**Table 5:**
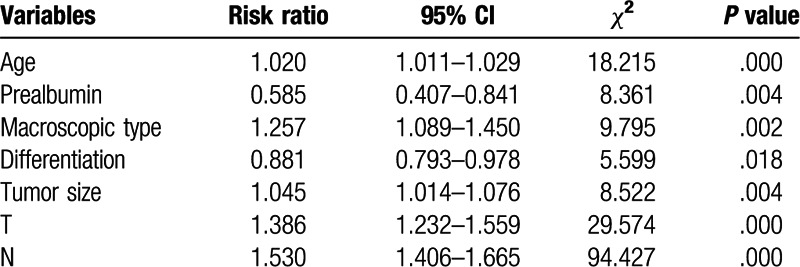
Multivariate analysis of prognostic factors in 857 patients with gastric cancer (includes only patients with radical resection).

## 4 Discussion

Serum prealbumin is a negative acute-phase protein synthesized by the liver and is often used to evaluate the nutritional status of patients with malignant tumors.^[11–15]^ Prealbumin is a transport protein for thyroxine and a carrier for retinol-binding protein,^[16]^ with a half-life of approximately 2 days, which is significantly shorter than that of albumin (approximately 20 days).^[17,18]^ Many studies have shown that prealbumin is used to judge the complications of patients after surgery^[17,19–21]^ and the prognosis of patients with malignant tumors.^[22–25]^ Bae et al^[20]^ reported that preoperative prealbumin levels could be a useful marker for predicting complications after gastric surgery, especially infectious complications. Yu et al^[19]^ showed that patients with preoperative prealbumin levels ≤20 mg/dL had an increased risk for postoperative infections and intubation for >12 hours during cardiac surgery. Shimura et al^[22]^ selected 22.8 mg/dL as the cut-off prealbumin value (receiver operating characteristic [ROC] curve analysis) and found that the serum prealbumin level was an independent prognostic factor for GC patients (n = 30). In accordance with the above study, we determined that the optimal cut-off value of 140 mg/L had the maximum chi-square value related to disease-specific survival and indicated that the 5-year survival rate of patients with low prealbumin was significantly lower than that of patients with high prealbumin, demonstrating that the preoperative prealbumin level was an independent prognostic factor for patients with GC. Various cut-off prealbumin concentrations have been used by different researchers. Han et al^[23]^ selected 200 mg/L as the cut-off prealbumin value (ROC curve analysis) and confirmed that the preoperative prealbumin was an independent prognostic factor in patients with esophagogastric junction adenocarcinoma (n = 101). Zhang et al^[24]^ selected 180 mg/L as the cut-off prealbumin value (ROC curve analysis) and demonstrated that the preoperative prealbumin was an independent prognostic factor in patients with resected non-metastatic Siewert type II/III adenocarcinoma of the esophagogastric junction (n = 355). Esfahani et al^[25]^ selected 200 mg/L as the cut-off prealbumin value (ROC curve analysis) and showed that the prealbumin level was a predictive marker for metastasis in patients with inoperable GC (n = 71). Shen et al^[8]^ selected 180 mg/L as the cut-off prealbumin value (survival correlation analysis) and found that the preoperative prealbumin was an independent prognostic factor for patients with stage II/III GC who underwent gastrectomy (n = 731). The discrepancy in the prognostic prealbumin cut-off values among different studies may be related to the different research methods and patient selection processes. The results of our study, which included patients with tumors of stages and locations, were similar to previous study results. The prealbumin level plays an important role in judging the prognosis of patients with GC and can provide information to guide clinical practice. Regretfully, we did not record the complications of these patients.

In addition to the survival outcomes, patients with lower preoperative prealbumin levels had lower Hb levels, were older and had more proximal GC tumors than those with higher preoperative prealbumin levels. Moreover, patients with low preoperative prealbumin were more likely to have larger tumors, deeper invasion, more positive lymph nodes, and liver metastasis than those with high preoperative prealbumin. Our previous study^[27]^ found a significantly lower 5-year survival rate in proximal GC patients than in distal GC patients (28% vs 51%, respectively; *P* < .001). Furthermore, tumor size has been suggested to be a significant prognostic factor in GC.^[28]^ Additionally, N stage and T stage were recognized as predictors in GC patients. Low prealbumin levels can damage the immune system and inhibit cell-mediated immune function, leading to increased metastasis.^[29]^ All of the above aggressive characteristics lead to a poor prognosis in patients with low prealbumin levels.

The reasons for the strong association between low preoperative prealbumin levels and poor prognosis of GC patients are not yet clear. A possible mechanism may be the increased inflammatory status of *Helicobacter pylori* (*H pylori*).^[30]^ Infections with the human pathogen *H pylori* are closely associated with neoplastic transformation of the gastric epithelium.^[31]^ It has been established that patients with lower prealbumin levels have higher levels of high-sensitivity C-reactive protein (hs-CRP). As a negative acute-phase protein, the synthesis of prealbumin is suppressed in the inflammatory state,^[32]^ which mainly involves tumour necrosis factor alpha (TNF-α), interleukin-1 (IL-1), and IL-6, resulting in the increased generation of hs-CRP and decreased synthesis of prealbumin by the liver.^[33,34]^ Another possible underlying mechanism may be related to the effect of prealbumin on the transport of high-density lipoproteins (HDLs), in association with apolipoprotein A1. Liz et al^[35]^ believed that the presence of prealbumin affects the properties or stability of HDL particles. Prealbumin causes specific cleavage of apolipoprotein A1.^[36,37]^ Therefore, changes in prealbumin levels lead to changes in apolipoprotein A1 levels. Reductions in apolipoprotein A1 have been reported in GC and may be a new prognostic factor of GC.^[38,39]^ Therefore, the reduction in apolipoprotein A1 in GC may be related to low levels of prealbumin. In the future, we would like to further explore the underlying mechanism between prealbumin and apolipoprotein A1 levels in GC patients.

Our study lacked data on the administration of adjuvant chemotherapy because the extent of systemic adjuvant chemotherapy was uncertain for most patients. Another limitation was that the analyzed data in this study originated from a single center. In the future, we expect multi-center, large-scale collaborative research to prove the prognostic significance of prealbumin in GC patients.

## 5 Conclusions

In conclusion, the preoperative prealbumin level is an independent prognostic factor for GC patients. It is important to predict the prognosis of patients with GC. For patients with lower prealbumin levels, we recommend aggressive treatment during the perioperative period, which may improve the prognosis of these patients.

## Acknowledgments

The authors gratefully acknowledge the contribution of the study participants.

## Author contributions

**Conceptualization:** Yingwei Xue.

**Data curation:** Huiling Wang, Yingwei Xue.

**Formal analysis:** Hongliang Zu, Chunfeng Li.

**Methodology:** Hongliang Zu.

**Project administration:** Hongliang Zu, Yingwei Xue.

**Resources:** Chunfeng Li.

**Supervision:** Hongliang Zu.

**Writing – original draft:** Huiling Wang, Chunfeng Li.

**Writing – review & editing:** Huiling Wang.
